# Prevalence and acquisition rate of methicillin resistant *Staphylococcus aureus* (MRSA) in internal medicine wards at the University Hospital of Geneva (HUG)

**DOI:** 10.1186/1753-6561-5-S6-P7

**Published:** 2011-06-29

**Authors:** T Koessler, J Pasricha, V Camus, G Renzi, S Harbarth, J Schrenzel, A Perrier, D Pittet, A Iten

**Affiliations:** 1 Department of Internal Medicine, University Hospital of Geneva, Geneva, Switzerland; 2 Department of Infection Control, Geneva, Switzerland; 3 Central Laboratory of Bacteriology, University Hospital of Geneva, Geneva, Switzerland

## Introduction / objectives

We aimed to determine the prevalence and acquisition rate of MRSA among patients admitted to internal medicine ward at HUG.

## Methods

Patients consecutively admitted to 13 medical wards from March-June 2010 were screened for MRSA using pooled axilla and groin swabs within 48 h of admission and 36 h of discharge.

## Results

Among 1967 patients, swabs were collected in 1740 (88%) at admission and 712 (36%) at discharge; with 687 (35%) having both. Mean age was 64 years; 58% were male. 4.8% (84/1740) of patients were MRSA positive on admission, of which 79% -for whom data were available (43/54)- had been MRSA positive on screening in the previous 6 months. Of patients who were not MRSA positive at admission, 5.8% (29/496) had previous carriage. MRSA carriage at admission was associated with age (p=0.0016, Wilcoxon rank sum test) and a positive MRSA swab in the previous 6 months (χ^2^ 233, p<0.0001). 3.8% (27/710) of patients acquired MRSA during hospital stay - having a positive MRSA swab on discharge but not on admission. By Wilcoxon rank sum test, MRSA acquisition was associated with length of hospital stay (p=0.0009) and age (p=0.0087). No association was found between MRSA carriage or acquisition and sex, provenance, antibiotic use, requirement of intensive or high dependency care and type of medical ward.

## Conclusion

4.8% of patients admitted to general medical wards at our hospital were MRSA positive, the majority of these patients had previous MRSA carriage. 3.8% of patients acquired MRSA during hospital stay.

## Disclosure of interest

None declared.

